# Prognostic significance of sarcopenia and severe vitamin D deficiency in patients with cirrhosis

**DOI:** 10.1002/jgh3.12900

**Published:** 2023-04-14

**Authors:** Chisato Saeki, Tomoya Kanai, Kaoru Ueda, Masanori Nakano, Tsunekazu Oikawa, Yuichi Torisu, Masayuki Saruta, Akihito Tsubota

**Affiliations:** ^1^ Division of Gastroenterology and Hepatology, Department of Internal Medicine The Jikei University School of Medicine Tokyo Japan; ^2^ Division of Gastroenterology, Department of Internal Medicine Fuji City General Hospital Shizuoka Japan; ^3^ Research Center for Medical Science The Jikei University School of Medicine Tokyo Japan

**Keywords:** cirrhosis, prognosis, sarcopenia, severe vitamin D deficiency

## Abstract

**Background and Aim:**

Sarcopenia and severe vitamin D deficiency are associated with malnutrition and poor prognosis. We investigated the impact of the comorbidity of Child–Pugh (CP) class B/C cirrhosis and the aforementioned complications on the prognosis of patients with cirrhosis.

**Methods:**

We retrospectively evaluated 104 patients with cirrhosis. The cumulative survival rates were compared between patients with and without both or either of these disease conditions: CP class B/C and complications (sarcopenia or severe vitamin D deficiency). Sarcopenia was diagnosed according to the Japan Society of Hepatology criteria. Severe vitamin D deficiency was defined as levels of 25‐hydroxyvitamin D <10 ng/mL in serum.

**Results:**

The prevalence of CP class B/C, sarcopenia, and severe vitamin D deficiency was 26.9%, 38.5%, and 24.0%, respectively. Patients with both CP class B/C and sarcopenia had significantly lower survival rates than those without both (hazard ratio [HR] = 6.101; *P* < 0.001) and with either condition (HR = 6.137; *P* = 0.001). Similarly, patients with both CP class B/C and severe vitamin D deficiency or with either condition had significantly lower survival rates than those without both conditions (HR = 8.135 or 3.189; *P* < 0.001 or =0.025, respectively). CP class B/C (HR = 3.354; *P* = 0.006) and severe vitamin D deficiency (HR = 2.445; *P* = 0.044) were independent prognostic factors.

**Conclusions:**

The coexistence of CP class B/C and sarcopenia or severe vitamin D deficiency worsened the prognosis of patients with cirrhosis. Nutritional assessments such as sarcopenia and vitamin D status should be considered to better evaluate disease conditions and patient prognosis.

## Graphical abstract

The coexistence of Child‐Pugh class B/C and sarcopenia or severe vitamin D deficiency worsened the prognosis of patients with cirrhosis.

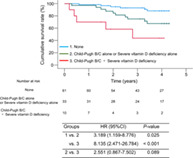

## Introduction

Cirrhosis is the end stage of progressive liver fibrosis in chronic liver disease (CLD) and is one of the leading causes of morbidity and mortality worldwide.^1^ The Child–Pugh (CP) classification, which was initially developed to predict surgical outcomes, has been widely used to assess liver functional reserve in patients with cirrhosis. It comprises five items: serum albumin, total bilirubin, prothrombin time, ascites degree, and encephalopathy grade.^2^ This scoring system can predict the prognosis of cirrhosis: patients with CP class A have a better prognosis than those with CP class B/C.^3^ However, its major limitation is that it does not assess patients' nutritional status, which has a critical impact on mortality in patients with cirrhosis.^4^, ^5^

Sarcopenia, which is characterized by the progressive loss of skeletal muscle mass, strength, and function, is a common complication of cirrhosis.^6^ Protein‐energy malnutrition, macro and micronutrient deficiencies, cirrhosis‐related factors (such as reduced branched‐chain amino acids [BCAAs], hyperammonemia, and increased inflammatory cytokine levels), and physical inactivity are involved in sarcopenia development.^7^, ^8^, ^9^ Sarcopenia is also an independent risk factor for poor prognosis in patients with cirrhosis.^10^, ^11^, ^12^, ^13^ A recent meta‐analysis of 22 studies on cirrhosis in 6965 patients revealed that sarcopenia increases the mortality risk (adjusted hazard ratio [HR] = 2.3).^14^ Therefore, physicians should determine whether patients with cirrhosis have sarcopenia to better predict their prognosis.

Vitamin D is a secosteroid hormone that is involved in bone metabolism and muscle cell growth and function.^15^ CLD is frequently complicated by vitamin D deficiency (25‐hydroxyvitamin D [25(OH)D] <20 ng/mL) or severe vitamin D deficiency (<10 ng/mL), with a prevalence of 71–87% and 32–53%, respectively.^16^, ^17^, ^18^, ^19^, ^20^ In our previous study on patients with CLD, lower 25(OH)D levels, especially severe vitamin D deficiency status (≤10.5 ng/mL), were significantly associated with sarcopenia.^18^ Notably, severe vitamin D deficiency has been widely reported as an independent risk factor for a poor prognosis in patients with CLD.^19^, ^20^ Therefore, the comorbidity of impaired liver functional reserve and sarcopenia or severe vitamin D deficiency may worsen the prognosis of patients with cirrhosis. However, no study has analyzed these factors together in the same population.

Hence, this study aimed to investigate the relationship between CP class B/C cirrhosis complicated by sarcopenia or severe vitamin D deficiency and the prognosis of patients with cirrhosis.

## Materials and Methods

### Study participants

This retrospective study included 104 consecutive patients with cirrhosis who visited the Jikei University School of Medicine (Tokyo, Japan) and Fuji City General Hospital (Shizuoka, Japan) between 2017 and 2020. The inclusion criteria were patients (i) diagnosed with cirrhosis and (ii) with available data on skeletal muscle mass index (SMI, measured using bioimpedance analysis [InBody S10; InBody, Seoul, Korea]) and grip strength (measured using a dynamometer [T.K.K5401 GRIP‐D; Takei Scientific Instruments, Niigata, Japan]). The exclusion criteria were (i) vitamin D supplementation within the past 12 months; (ii) alcoholic liver disease resulting from heavy alcohol consumption, which causes extreme undernutrition (including severe vitamin D deficiency); (iii) pre‐existence of uncontrollable malignancy (including hepatocellular carcinoma [HCC]); and (iv) application of pacemaker, implants, or massive ascites. Cirrhosis was diagnosed according to laboratory tests and imaging/endoscopic findings, such as the presence of ascites, liver deformation, surface nodularity, and esophageal/gastric varices. The model for end‐stage liver disease (MELD) score and CP classification were used to assess liver functional reserve.^2^, ^21^ HCC was diagnosed according to medical imaging, such as ultrasonography, computed tomography, and magnetic resonance imaging, in accordance with the HCC guidelines of the American Association for the Study of Liver Diseases.^22^ Patients with HCC treatment history or controllable HCC at entry were considered as having HCC, and patients who underwent liver transplant for liver failure during the follow‐up period were regarded as mortality and censored cases.

This study complied with the 2013 Declaration of Helsinki and obtained approval from the ethics committees of the Jikei University School of Medicine (approval No.: 34‐021) and Fuji City General Hospital (approval No.: 279).

### Sarcopenia diagnosis

Sarcopenia was diagnosed according to the revised criteria proposed by the Japan Society of Hepatology (second edition).^23^ The reference values for reduced SMI and handgrip strength are <7.0 kg/m^2^ and <28 kg for men and <5.7 kg/m^2^ and <18 kg for women, respectively.

### Laboratory assessments

Serum albumin, total bilirubin, sodium, and prothrombin time were measured using standard laboratory methods. Serum 25(OH)D, which is the main circulating form of vitamin D and reflects vitamin D status, was measured using a chemiluminescent immunoassay (Hitachi Chemical Diagnostics Systems, Tokyo). Vitamin D deficiency and severe vitamin D deficiency were defined as serum 25(OH)D levels of <20 and <10 ng/mL, respectively.^19^, ^20^, ^24^

### Statistical analysis

Categorical variables are presented as numbers and percentages, whereas continuous variables are expressed as medians and interquartile ranges. The significance of differences between two groups was assessed using the chi‐squared test for the categorical variables and the Mann–Whitney *U*‐test for the continuous variables. The cumulative survival rates were estimated using the Kaplan–Meier method, and the differences between groups were compared using the log‐rank test. Univariate and multiple Cox proportional hazards models were used to identify significant and independent factors associated with mortality. SPSS Statistics version 27 (IBM Japan, Tokyo) was used for all statistical analyses. A *P‐*value of <0.05 was considered statistically significant.

## Results

### Patient characteristics

A flow diagram of patients included in this study is shown in Figure S1, Supporting information. Of the 158 patients with cirrhosis evaluated for eligibility, 54 met the exclusion criteria and the remaining 104 patients were enrolled in this study. Table 1 summarizes the baseline characteristics of all patients. The median age was 73.0 (63.0–79.0) years, and 58 (55.8%) of them were men. The median 25(OH) D level was 13.7 (10.2–17.5) ng/mL. The median MELD score was 8.0 (7.0–10.0). The prevalence of CP class B/C and HCC was 26.9% (28/104) and 47.1% (49/104), respectively. The proportion of patients with ascites was 26.0% (27/104).

**Table 1:** Comparison of clinical characteristics between patients with and without sarcopenia

Variable	All patients	Sarcopenia	Non‐sarcopenia	*P‐*value
Patients, *n* (%)	104	40 (38.5)	64 (61.5)	—
Men, *n* (%)	58 (55.8)	19 (47.5)	39 (60.9)	0.179
Age (years)	73.0 (63.0–79.0)	76.0 (72.3–80.0)	70.5 (61.0–78.0)	0.005
Etiology				
HBV/HCV/other, *n*	16/50/38	3/27/10	13/23/28	0.006
Child‐Pugh B/C, *n* (%)	28 (26.9)	15 (37.5)	13 (20.3)	0.055
MELD score	8.0 (7.0–10.0)	8.0 (7.0–10.8)	8.0 (7.0–10.0)	0.367
Total bilirubin (mg/dL)	0.8 (0.6–1.2)	0.8 (0.5–1.3)	0.8 (0.6–1.0)	0.492
Albumin (g/dL)	3.9 (3.4–4.3)	3.9 (3.1–4.3)	4.0 (3.6–4.4)	0.087
Sodium (mEq/L)	140 (139–141)	140 (137–142)	140 (139–141)	0.471
Prothrombin time	85 (71–100)	84 (68–100)	85 (73–98)	0.848
25(OH)D (ng/mL)	13.7 (10.2–17.5)	11.3 (8.3–14.7)	14.7 (11.1–18.7)	0.002
Vitamin D deficiency, *n* (%)	91 (87.5)	37 (92.5)	54 (84.4)	0.223
Severe vitamin D deficiency, *n* (%)	25 (24.0)	16 (40.0)	9 (14.1)	0.003
HCC, *n* (%)	49 (47.1)	20 (50.0)	29 (45.3)	0.641

Continuous variables are shown as median (interquartile range). Statistical analysis was performed using the chi‐squared test or the Mann–Whitney *U*‐test, as appropriate.

25(OH)D, 25‐hydroxyvitamin D; HBV, hepatitis B virus; HCC, hepatocellular carcinoma; HCV, hepatitis C virus; MELD, model for end‐stage liver disease.

### Clinical characteristics of patients with sarcopenia or severe vitamin D deficiency

The prevalence of sarcopenia was 38.5% (40/104, Table 1). Patients with sarcopenia (sarcopenia group) were significantly older (*P* = 0.005) than those without sarcopenia (non‐sarcopenia group). The prevalence of CP class B/C showed a marginally significant difference between these two groups (*P* = 0.055).

The sarcopenia group had significantly lower serum 25(OH) D levels than the non‐sarcopenia group (*P* = 0.002). This overall cohort included 91 (87.5%) patients with vitamin D deficiency and 25 (24.0%) patients with severe vitamin D deficiency (Tables 1 and 2). Notably, the sarcopenia group had a significantly higher prevalence of severe vitamin D deficiency (but not vitamin D deficiency) than the non‐sarcopenia group (40.0% *vs* 14.1%, *P* = 0.003, Table 1). Conversely, the severe vitamin D deficiency group had a significantly higher prevalence of sarcopenia than the nonsevere vitamin D deficiency group (64.0% *vs* 30.4%, *P* = 0.003, Table 2). These findings suggest that patients with sarcopenia may be susceptible to severe vitamin D deficiency, and vice versa.

**Table 2:** Comparison of clinical characteristics between patients with and without severe vitamin D deficiency

Variable	Severe vitamin D deficiency	Non‐severe vitamin D deficiency	*P‐*value
Patients, *n* (%)	25 (24.0)	79 (76.0)	—
Man, *n* (%)	12 (48.0)	46 (58.2)	0.369
Age (years)	73.0 (61.5–78.0)	73.0 (63.0–79.0)	0.550
Etiology			
HBV/HCV/other, *n*	1/12/12	15/38/26	0.138
Child‐Pugh B/C, *n* (%)	10 (40.0)	18 (22.8)	0.091
MELD score	9.0 (8.0–12.5)	8.0 (7.0–10.0)	0.044
Total bilirubin (mg/dL)	0.9 (0.5–1.8)	0.8 (0.6–1.0)	0.149
Albumin (g/dL)	3.7 (3.0–4.5)	4.0 (3.5–4.3)	0.250
Sodium (mEq/L)	140 (137–141)	140 (139–141)	0.180
Prothrombin time	77 (68–89)	86 (74–100)	0.060
25(OH)D (ng/mL)	7.8 (6.6–8.8)	15.1 (13.0–18.7)	< 0.001
Sarcopenia, *n* (%)	16 (64.0)	24 (30.4)	0.003
HCC, *n* (%)	10 (40.0)	39 (49.4)	0.413

Continuous variables are shown as median (interquartile range). Statistical analysis was performed using the chi‐squared test or the Mann–Whitney *U*‐test, as appropriate.

25(OH)D, 25‐hydroxyvitamin D; HBV, hepatitis B virus; HCC, hepatocellular carcinoma; HCV, hepatitis C virus; MELD, model for end‐stage liver disease.

### Impact of CP class B/C cirrhosis, sarcopenia, and severe vitamin D deficiency on survival

**Figure 1:**
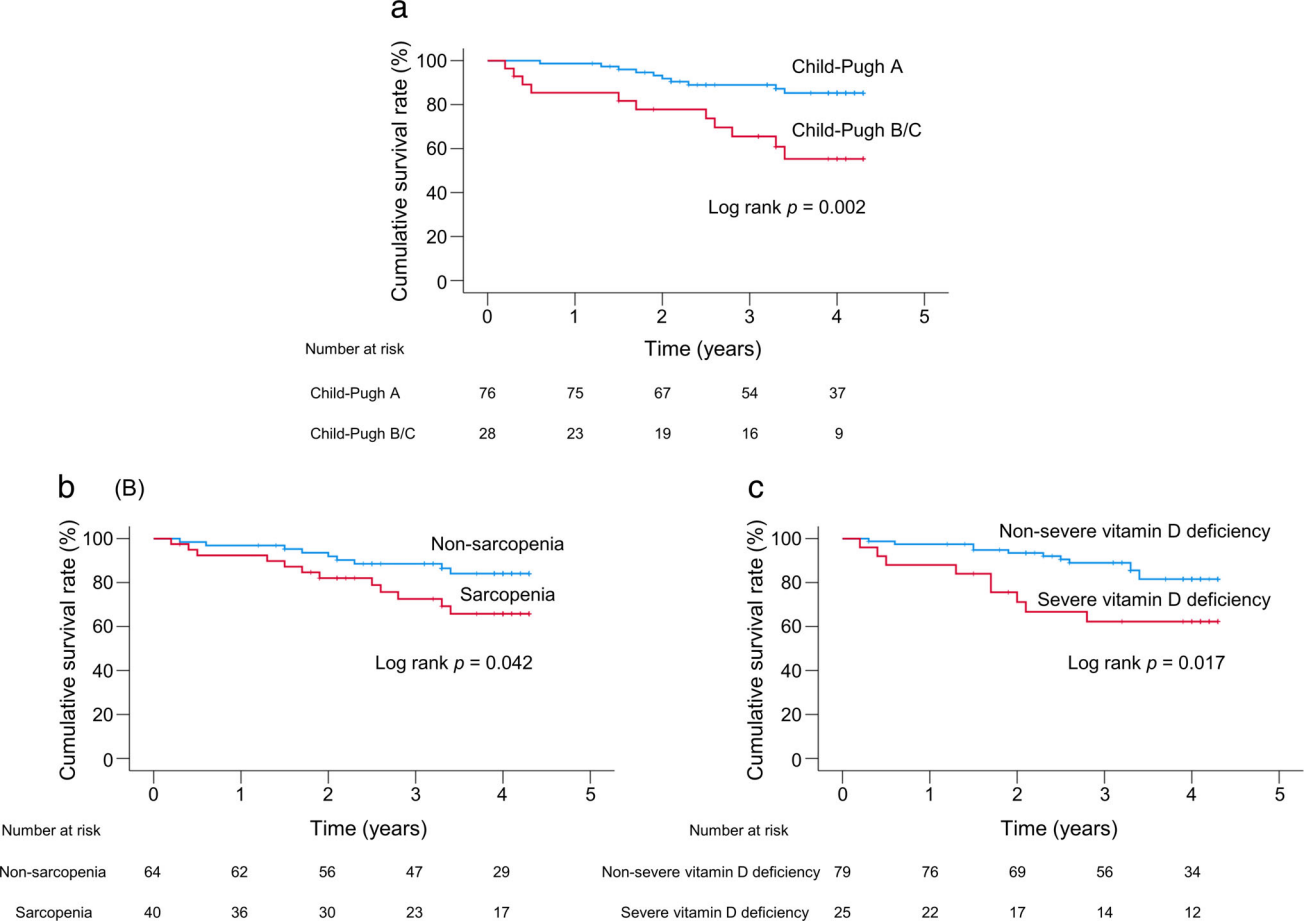
Cumulative survival rates in patients with and without Child–Pugh class B/C (a), sarcopenia (b), and severe vitamin D deficiency (c).

The median observation period was 45.5 (28.3–49.0) months. During the follow‐up period, 21 (20.2%) patients died of liver‐disease‐related events, including liver failure (*n* = 9), liver transplantation (*n* = 1), HCC (*n* = 8), and rupture of esophageal varices (*n* = 3) (Fig. S1). The cumulative survival rates were significantly lower in the CP class B/C group than in the CP class A group (*P* = 0.002, Fig. 1a). The 1‐ and 3‐year survival rates were 85.4% and 65.5% in the CP class B/C group and 98.7% and 89.0% in the CP class A group, respectively.

The cumulative survival rates in the sarcopenia group were significantly lower than in the non‐sarcopenia group (*P* = 0.042, Fig. 1b). The 1‐ and 3‐year survival rates were 92.4% and 72.6% in the sarcopenia group and 96.9% and 88.5% in the non‐sarcopenia group, respectively.

Regarding the difference in prognosis between groups with severe vitamin D deficiency and nonsevere vitamin D deficiency, the former had significantly lower cumulative survival rates (*P* = 0.017, Fig. 1c). The 1‐ and 3‐year survival rates were 88.0% and 62.3% in the severe vitamin D deficiency group and 97.5% and 89.0% in the nonsevere vitamin D deficiency group, respectively.

### Impact of comorbidity of CP class B/C cirrhosis and sarcopenia or severe vitamin D deficiency on survival

**Figure 2:**
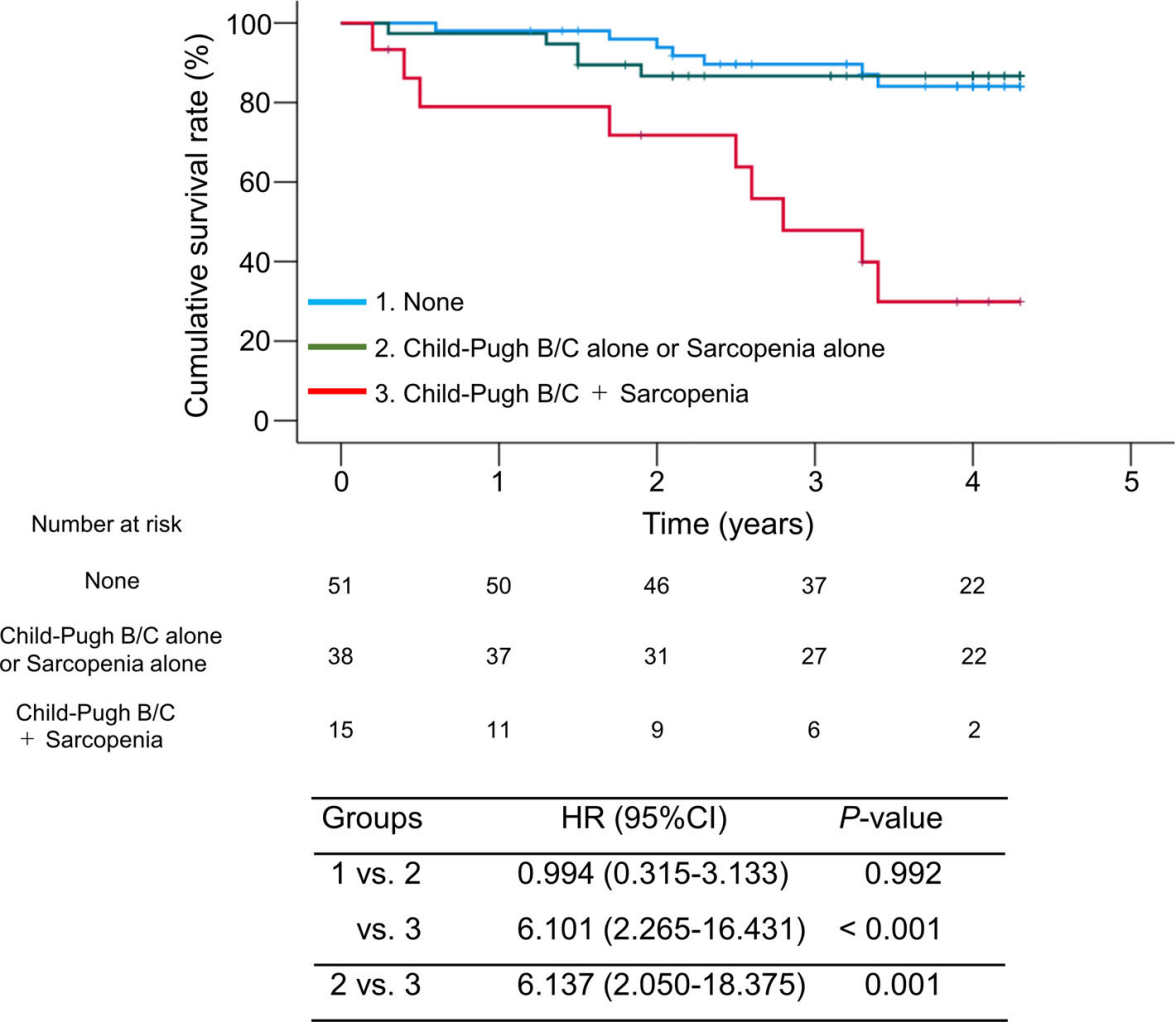
Prognostic significance of Child–Pugh class B/C cirrhosis and sarcopenia as comorbidities. Patients with both conditions had significantly lower cumulative survival rates than those without both (*P* < 0.001) or those with either condition (*P* = 0.001).

We classified the patients into three groups according to the presence or absence of CP class B/C cirrhosis and/or sarcopenia: (i) patients without both CP class B/C and sarcopenia (*n* = 51); (ii) patients with CP class B/C alone or sarcopenia alone (*n* = 38); and (iii) patients with both CP class B/C and sarcopenia (*n* = 15). Patients with both conditions had significantly lower cumulative survival rates than those without both conditions (HR = 6.101; 95% confidence interval [CI]: 2.265–16.431; *P* < 0.001) and those with either condition (HR = 6.137; 95% CI: 2.050–18.375; *P* = 0.001) (Fig. 2).

**Figure 3:**
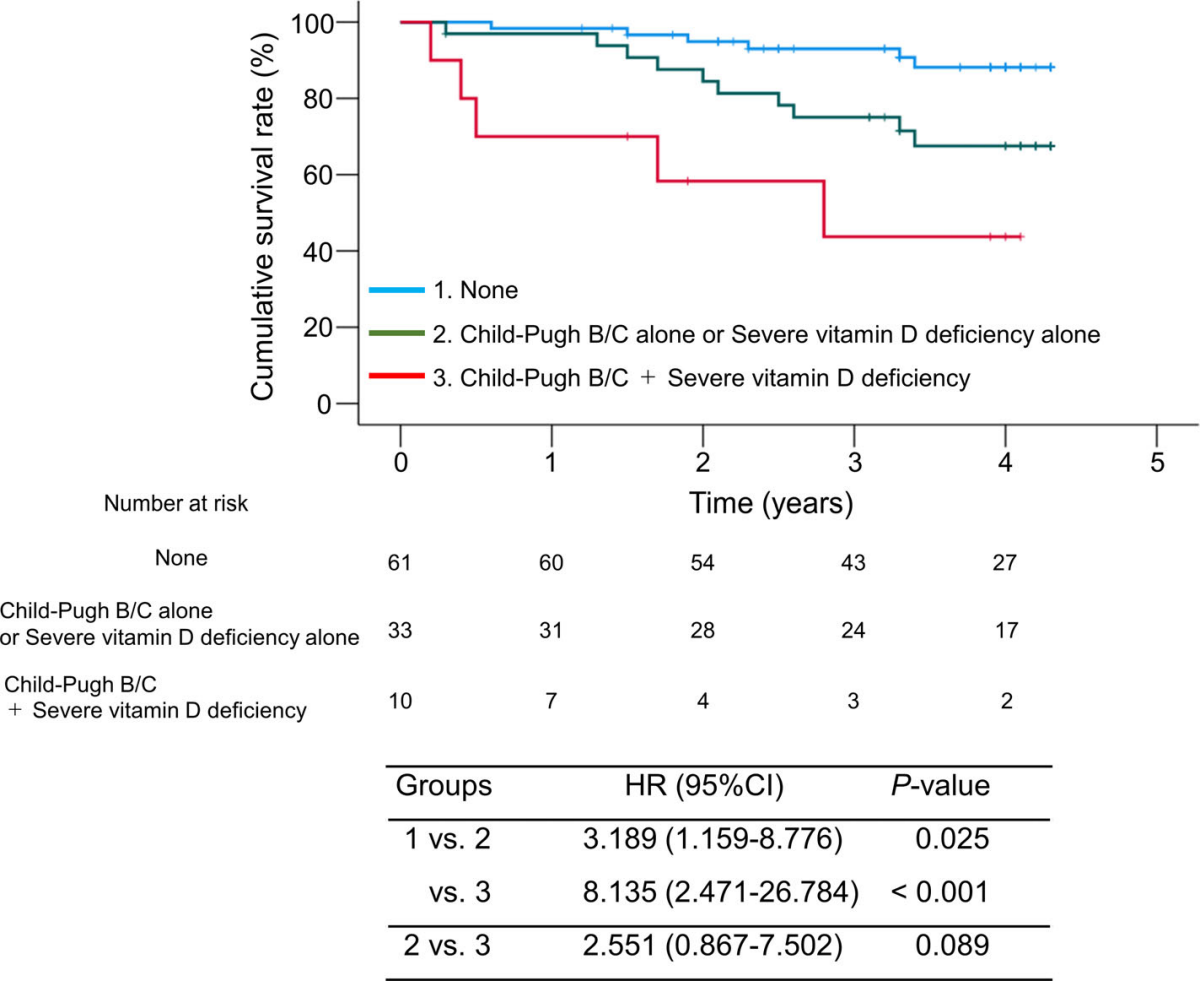
Prognostic significance of Child–Pugh class B/C cirrhosis and severe vitamin D deficiency as comorbidities. Patients with either condition or both conditions had significantly lower cumulative survival rates than those without both conditions (*P* = 0.025 or <0.001, respectively). A marginally significant difference was observed in the cumulative survival rates between patients with either condition and both conditions (*P* = 0.089).

We also classified the patients into three groups according to the presence or absence of CP class B/C cirrhosis and/or severe vitamin D deficiency: (i) patients without both CP class B/C and severe vitamin D deficiency (*n* = 61); (ii) patients with CP class B/C alone or severe vitamin D deficiency alone (*n* = 33); and (iii) patients with both CP class B/C and severe vitamin D deficiency (*n* = 10). Patients with either condition or both conditions had significantly lower cumulative survival rates than those without both conditions (HR = 3.189 or 8.135; 95% CI: 1.159–8.776 or 2.471–26.784; *P* = 0.025 or <0.001, respectively) (Fig. 3). A marginally significant difference was found in the cumulative survival rates between patients with either condition and both conditions (HR = 2.551; 95% CI, 0.867–7.502; *P* = 0.089).

### Prognostic factors in patients with cirrhosis

In the univariate analysis, CP class B/C, MELD score, sodium, sarcopenia, and severe vitamin D deficiency (but not vitamin D deficiency) were significantly associated with mortality (Table S1, Supporting information). Cox proportional hazards regression analysis identified CP class B/C (HR = 3.354; 95% CI: 1.416–7.946; *P* = 0.006) and severe vitamin D deficiency (HR = 2.445; 95% CI: 1.023–5.843; *P* = 0.044) as significant and independent prognostic factors in patients with cirrhosis (Table 3).

**Table 3:** Significant factors associated with mortality in patients with liver cirrhosis

Variable	Univariate		Multivariate	
	HR (95% CI)	*P*‐value	HR (95% CI)	*P*‐value
Child–Pugh B/C	3.625 (1.538–8.545)	0.003	3.354 (1.416–7.946)	0.006
MELD score	1.132 (1.003–1.278)	0.045	—	—
Sodium (mEq/L)	0.825 (0.720–0.944)	0.005	—	—
Sarcopenia	2.382 (1.003–5.656)	0.049	—	—
Severe vitamin D deficiency	2.736 (1.152–6.499)	0.023	2.445 (1.023–5.843)	0.044

CI, confidence interval; HR, hazard ratio; MELD, model for end‐stage liver disease.

## Discussion

Malnutrition is a serious complication in patients with cirrhosis and is associated with negative outcomes, such as ascites, infections, and hepatic encephalopathy, leading to a poor prognosis.^4^, ^5^ Therefore, nutritional assessment should be more thoroughly considered when evaluating disease conditions and patient prognosis. Our study investigated the impact of the comorbidity of CP class B/C cirrhosis and sarcopenia/severe vitamin D deficiency (which are closely associated with nutrient states) on the prognosis of patients with cirrhosis. As a result, patients with either condition or both these conditions had lower survival rates than those without these conditions. Notably, sarcopenia and severe vitamin D deficiency worsened the prognosis of patients with CP class B/C.

The pathophysiological mechanisms of malnutrition in cirrhosis are multifactorial and complex. For instance, reduced oral intake of macro/micronutrients caused by anorexigenic and orexigenic hormone imbalance, nutrient maldigestion/malabsorption, decreased hepatic glucose production and glycogen stores, decreased BCAAs caused by energy production and ammonia detoxification, and increased proinflammatory cytokine levels are involved in malnutrition, leading to sarcopenia.^8^, ^9^, ^25^ In one study on CLD, patients with malnutrition, as assessed by the Global Leadership Initiative on Malnutrition criteria, had significantly higher prevalence of sarcopenia and mortality than well‐nourished patients.^26^ In another study on cirrhosis, sarcopenia, as well as decompensated cirrhosis and HCC stage 3/4, was an independent prognostic factor.^13^ Additionally, patients with sarcopenia had a higher incidence of cirrhosis‐related complications, such as ascites, spontaneous bacterial peritonitis, hepatic encephalopathy, and esophageal/gastric varices.^12^ In the other study on cirrhosis, sarcopenia, CP class B/C, and BCAA supplementation were independently associated with survival rates.^10^ Long‐term BCAA supplementation significantly improved sarcopenic parameters (such as muscle strength, muscle mass, and muscle function) and the prognosis of patients with sarcopenia.^10^, ^27^ These results suggest that nutrient states and sarcopenia are closely associated with cirrhosis and its prognosis, and that early nutritional intervention is crucial to prevent the development or progression of malnutrition and sarcopenia and improve the prognosis.

Our univariate analyses identified CP class B/C, severe vitamin D deficiency, and sarcopenia as significant prognostic factors; however, in multivariate analysis, only the first two factors remained significant. Given that low 25(OH)D levels, especially very low levels, are closely associated with sarcopenia,^17^, ^18^ severe vitamin D deficiency may negate sarcopenia as an independent prognostic factor. Vitamin D deficiency is highly prevalent in patients with CLD.^16^, ^17^, ^18^, ^19^, ^20^ Indeed, 87.5% of this study cohort had vitamin D deficiency; therefore we focused on severe vitamin D deficiency (but not vitamin D deficiency). In a recent nonlinear Mendelian randomization study of UK Biobank participants, genetically predicted 25 (OH)D levels had an L‐shaped association with all‐cause mortality (cardiovascular, malignant, and respiratory diseases), and the mortality risk decreased sharply as the 25(OH) D levels increased (until 20 ng/mL).^28^ Participants with 25(OH)D levels of 10 ng/mL had a 25% higher mortality risk than those with the levels of 20 ng/mL. In one study of patients with cirrhosis, severe vitamin D deficiency, CP class B/C, MELD score ≥15, active HCC, and α‐fetoprotein (AFP) >10 ng/mL were associated with survival rates; however, only severe vitamin D deficiency and CP class B/C were identified as independent prognostic factors in the multivariate analysis.^19^ In another study of patients with alcoholic liver disease, severe vitamin D deficiency was independently associated with cirrhosis and survival rates at 1 year of the study period.^20^ In yet another study of patients with HCC, severe vitamin D deficiency was associated with mortality independent of the MELD score and high AFP levels (>400 ng/mL).^29^ These results indicate that severe vitamin D deficiency, regardless of the etiology and condition of underlying diseases, has a critical impact on the prognosis. The definition of vitamin D deficiency or the cut‐off value of vitamin D level for predicting the prognosis of patients with cirrhosis may need to be reconsidered.

The CP classification is generally used to evaluate liver functional reserve. Patients with CP class B/C have lower survival rates than those with CP class A.^3^ However, this scoring system does not include nutritional assessments. The present study is the first to investigate the impact of incorporating severe vitamin D deficiency in the CP classification on patients' prognosis. Patients with CP class B/C and severe vitamin D deficiency had a significantly worse prognosis than those with neither or either of these factors. In a previous study of patients with cirrhosis, the prevalence of severe vitamin D deficiency increased stepwise with decreasing liver functional reserve (14% in CP class A, 39% in CP class B, and 47% in CP class C).^30^ Given that vitamin D deficiency is associated with increased inflammatory cytokine levels, immune system dysfunction, bacterial translocation, increased portal hypertension, and advanced liver disease,^31^, ^32^, ^33^ patients with severe vitamin D deficiency may be more susceptible to liver‐disease‐related events, such as infections, spontaneous bacterial peritonitis, and esophageal varix rupture, leading to a poor prognosis. Meanwhile, our study revealed that sarcopenia concomitant with CP class B/C also decreased the survival rates. Reportedly, patients with CP class A and B combined with sarcopenia have lower survival rates than those without sarcopenia.^11^ Additionally, patients with CP class C or MELD score >14 combined with sarcopenia had lower 2‐year survival rates than those without sarcopenia.^12^ A meta‐analysis revealed that sarcopenia as comorbidity increases the mortality risk regardless of the severity of liver functional reserve (MELD scores: <15 or ≥15).^14^ Collectively, incorporating nutrition‐related factors such as severe vitamin D deficiency and sarcopenia into the conventional scoring systems for assessing liver functional reserve can help achieve better estimation of the prognosis of patients with cirrhosis.

This study has some limitations. First, the sample size was not large enough to evaluate the impact of sarcopenia and severe vitamin D deficiency on the prognosis of stratified subgroup patients. Thus, a large‐scale study is required to establish a prognostic prediction system that also considers the severity of vitamin D deficiency and sarcopenia. Second, we did not investigate patients' daily activities and nutritional intake, which might have influenced serum 25(OH)D levels, muscle mass, and muscle strength.

## Conclusions

CP class B/C, severe vitamin D deficiency, and sarcopenia decreased the survival rates of patients with cirrhosis, although the first two were independent prognostic predictors. The coexistence of CP class B/C cirrhosis and sarcopenia or severe vitamin D deficiency further increased the mortality. Nutritional assessments (such as sarcopenia and vitamin D status) in addition to the conventional scoring system for liver functional reserve are crucial to better evaluate disease conditions and patients' prognosis.

## Supporting information


**Figure S1.** Flow diagram of patients included in this study.

**Table S1.** Univariate analysis of factors associated with mortality.
