# Supplementary figures and images for: Prognostic significance of sarcopenia and severe vitamin D deficiency in patients with cirrhosis
Source: JGH Open. 2023 Apr 14;7(5):351–7. doi: 10.1002/jgh3.12900 (PMC10230111; doi:10.1002/jgh3.12900)

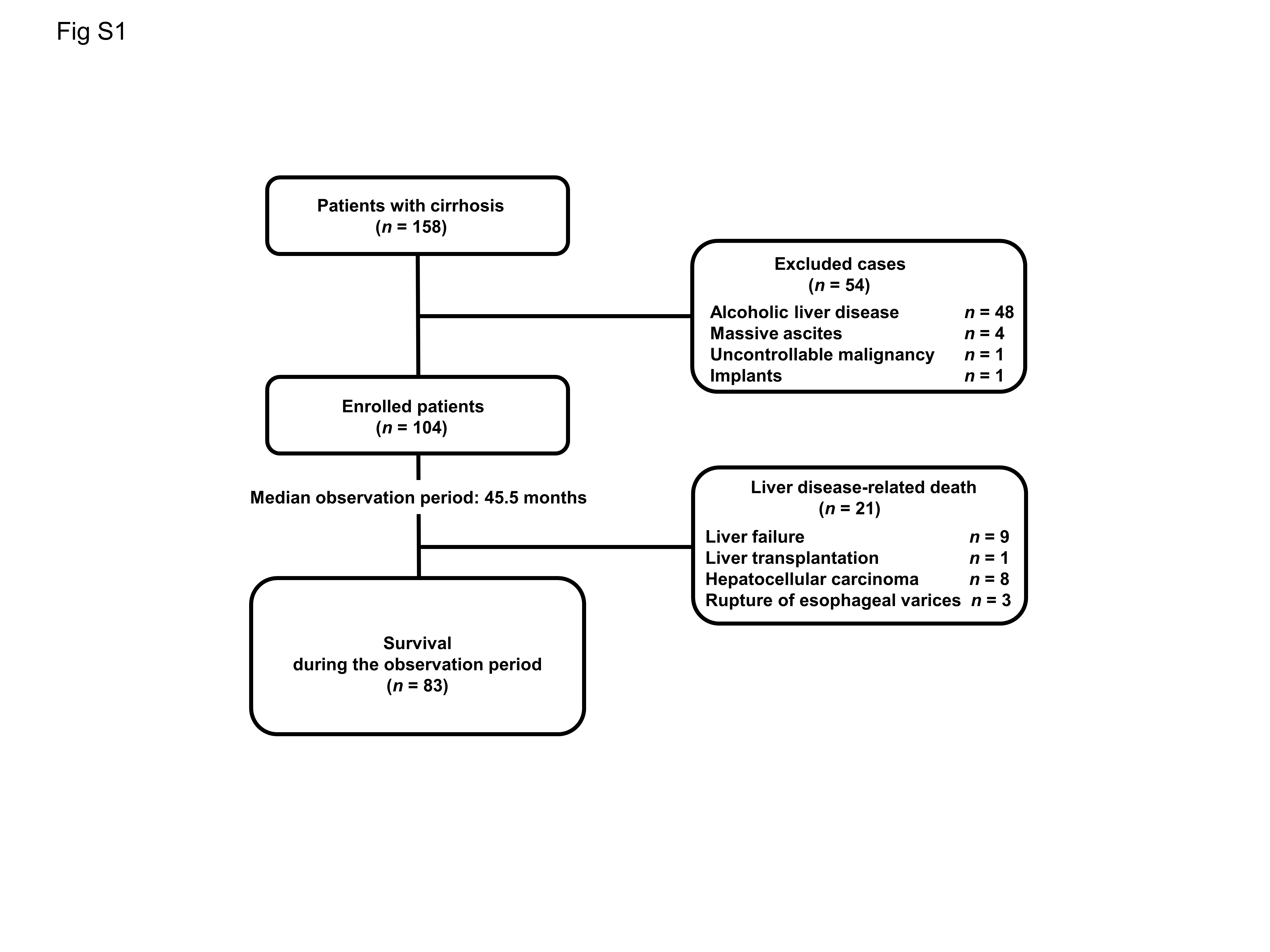

Supplement: Supplementary file 1 — Figure S1. Flow diagram of patients included in this study. [file JGH3-7-351-s002.tif]
