# Supplementary material for: Prognostic significance of sarcopenia and severe vitamin D deficiency in patients with cirrhosis
Source: JGH Open. 2023 Apr 14;7(5):351–7. doi: 10.1002/jgh3.12900 (PMC10230111; doi:10.1002/jgh3.12900)
Supplement: Supplementary file 2 — Table S1. Univariate analysis of factors associated with mortality. [file JGH3-7-351-s001.docx]

**Table S1. Univariate analysis of factors associated with mortality**

| Variable | HR (95% CI) | *p*-value |
| --- | --- | --- |
| Gender (Man) | 1.702 (0.687–4.219) | 0.231 |
| Age (years) | 0.994 (0.956–1.034) | 0.759 |
| Etiology | 1.431 (0.759–2.699) | 0.268 |
| Child-Pugh B/C | 3.625 (1.538–8.545) | 0.003 |
| MELD score | 1.132 (1.003–1.278) | 0.045 |
| Sodium (mEq/L) | 0.825 (0.720–0.944) | 0.005 |
| Sarcopenia | 2.382 (1.003–5.656) | 0.049 |
| Vitamin D deficiency | 25.255 (0.114–5618.231) | 0.241 |
| Severe vitamin D deficiency | 2.736 (1.152–6.499) | 0.023 |
| HCC | 1.656 (0.697–3.932) | 0.253 |

CI, confidence interval; HCC, hepatocellular carcinoma; HR, hazard ratio; MELD, model for end-stage liver disease.
